# Patterns and Determinants of Complementary and Alternative Medicine Practitioner Use among Adults with Diabetes in Queensland, Australia

**DOI:** 10.1155/2012/659419

**Published:** 2012-08-07

**Authors:** Chi-Wai Lui, Jo Dower, Maria Donald, Joseph R. Coll

**Affiliations:** School of Population Health, The University of Queensland, Herston Road, Brisbane, QLD 4006, Australia

## Abstract

There is evidence that complementary and alternative medicine (CAM) use is common among people with diabetes. The role of CAM in the treatment or management of diabetes is an emerging health issue given the potential side effects and benefits associated with the use of this kind of medicine. This paper examined patterns and determinants of CAM practitioner use in Queensland, Australia, using a large population-based sample of people with type 1 and type 2 diabetes. The study found that within a 12-month period, 7.7% of people with diabetes used the services of CAM practitioners alongside or as a complement to conventional health care service. Younger age, female gender, a higher education, having private health insurance, and engagement in preventive health behaviours are significant predictors of individuals who are more likely to visit a CAM practitioner. There was no significant difference in CAM practitioner use between people with type 1, type 2 insulin requiring, or type 2 noninsulin requiring diabetes. The findings highlight the need for further research on the role of CAM in the prevention and management of diabetes.

## 1. Introduction

Diabetes mellitus is one of the key global health challenges. In 2008, 347 million people worldwide were estimated to have diabetes [1]. In Australia, an estimated total of 898,800 Australians had been diagnosed with diabetes in 2007-2008. Among these individuals, 87,100 had been diagnosed with type 1 diabetes and 787,500 with type 2 diabetes [2]. It has been projected that 3 million Australians will be living with diabetes by 2025 as a result of recent increases in the incidence of obesity and declines in mortality [3]. 

Diabetes is a chronic debilitating illness that requires regular monitoring and control. The disease often has an adverse impact on the patients' quality of life. The management of diabetes requires the use of extensive health care services and is both physically and emotionally demanding [4, 5]. The degenerative nature of diabetes is such that the human and economic costs can increase dramatically with disease progression [6]. 

In recent years, there is a rising concern that many patients use complementary and alternative medicine (CAM) to cope with the daily challenge of diabetes and the multiple complications that are often associated with this condition [7–9]. The use of CAM may have side effects and can interact with conventional diabetes treatment [7]. There is evidence that CAM use is common among diabetes patients across different age groups [10–16] and cultures [17–21]. A recent literature review found that the prevalence of CAM use among people living with diabetes ranges from 17% to 73% [22]. The wide variation in reported prevalence likely reflects differences in research or sample design, definitions of CAM (practitioner and/or self-prescribed medication), or measurements of CAM use (e.g., lifetime use or use over the previous 12 months) employed by different research projects [22]. Current research findings suggest that nutritional supplements, herbal medicines, nutritional advice, spiritual healing, and relaxation techniques are the most widely consumed CAM therapies among diabetic populations [22].

Despite the rise in interest in the use of CAM among people with diabetes, the knowledge base for this issue remains limited. This is especially the case in Australia, since to date most studies on this topic have been conducted in North America. A recent study of the use of CAM for the treatment of chronic illness using the National Health Survey database found that about 4% of Australian adults used CAM for their treatment of diabetes in 2004-2005 [23]. This paper contributes to our understanding of CAM use among Australians living with type 1 and type 2 diabetes by examining the prevalence, profile, and predictors of CAM practitioner use in the state of Queensland, Australia, using a large population-based sample of people with diabetes. The study focuses broadly on diabetic patients' use of CAM practitioners for any purpose whether related or not to their diabetes management. 

## 2. Materials and Methods

### 2.1. Study Design and Sample

Data reported here are taken from the Living with Diabetes Study (LWDS), a five-year, prospective cohort study being conducted in the State of Queensland, Australia. The characteristics of the sample and methodology, including instrumentation, has been described in detail elsewhere [24], but briefly, the sample was recruited from the National Diabetes Services Scheme (NDSS), a government initiative that delivers diabetes-related products at subsidised prices to registrants and which covers up to 90% of the Australian population diagnosed with diabetes [2]. 

People were eligible to participate in the LWDS if they were aged 18 years or older, had been diagnosed with type 1 or type 2 diabetes (gestational diabetes was excluded), had a valid postal address recorded with the NDSS, and indicated on their NDSS registration that they were interested in receiving information about opportunities to participate in research. The sampling scheme intentionally oversampled in three areas of policy interest: an outer metropolitan area a new suburban development, and a coastal agricultural community. All eligible individuals from these three locations were invited to participate. In addition, a random sample of approximately one in six eligible individuals from the rest of the state was invited to participate. 

A sample of 14,439 NDSS registrants, out of a possible 155,874 who satisfied the eligibility criteria, was invited to participate in the LWDS study. Of these eligible responders, completed questionnaires and signed informed consent forms were returned by 3,951 participants, yielding a participation rate at baseline of 29%. The response rate for participants consenting to participate in the LWDS, while low, is consistent with research showing that participation rates in large cohort studies appear to be decreasing [25]. The response rate for the first follow-up (2009) survey, from which the results of this paper are drawn, was 88%  (*n* = 3,360). 

### 2.2. Measures

LWDS participants are mailed an annual self-report survey, which collects information on a range of health and well being issues. The survey covers eight areas including (i) demographic characteristics, (ii) clinical factors, including characteristics of diabetes, (iii) life-style-related factors, (iv) health-related quality of life, (v) self management, (vi) health care services utilisation, (vii) satisfaction with and quality of health care, and (viii) emotional well being. 

#### 2.2.1. CAM Practitioner Use

 Participants were asked to specify from a list which health professionals they had seen in the last 12 months. Respondents were defined as CAM users if they indicated they had consulted an alternative or complementary health practitioner during that time. Respondents who did not consult a CAM practitioner were classified as non-CAM users.

#### 2.2.2. Demographic Variables

 In addition to age and sex, participants were asked about the highest educational qualification they had completed, their household income, marital status, employment status and whether they had private health insurance. Postcode of residence was used to classify area of residence as urban, inner regional, or other. 

#### 2.2.3. Disease Characteristics

 Participants were classified according to the type of diabetes they had—type 1, type 2 insulin requiring, or type 2 noninsulin requiring. Self-reported age at diagnosis was used to determine duration of diabetes. Participants also reported their latest HbA1c result and the frequency of hypoglycaemic symptoms in the last month. Participants specified with which of a list of diabetes-related complications they had been diagnosed. Participants were also asked to specify with which of a list of comorbid conditions they had been diagnosed. 

#### 2.2.4. Lifestyle Behaviours

Participants reported their alcohol use, smoking status, physical activity in the last week, and dietary factors, including milk use, salt use, type of spread used on bread, and intake of fruit, vegetables, fish, and red meat. Body mass index (BMI) was calculated using the standard equation of weight divided by height squared [26]. 

#### 2.2.5. Health-Related Quality of Life

 Participants completed the EQ-5D [27], which is a short questionnaire with five questions on mobility, self-care, pain, social activities, and anxiety/depression. The Audit of Diabetes Dependent Quality of Life (ADDQoL) [28] was also completed as a disease-specific measure of the impact of diabetes on the patient's quality of life across 19 domains.

#### 2.2.6. Satisfaction with and Quality of Care

 Participants rated their satisfaction with their diabetes treatment as well as their overall satisfaction with the health care system. Participants were also asked about the ease of access to their main health care provider and to specialists as well as how long on average they had to wait for an appointment with their regular general practitioner. Patients were classified according to whether or not they were frequent attendees of their main health care provider, with frequent attendance defined as 12 or more self-reported visits in the past 12 months. Finally, respondents completed the Patient Assessmentof Chronic Illness Care (PACIC) scale [29], which measures the extent to which patients report receiving care in line with best practice regarding patient activation, delivery system design/decision support, goal setting/tailoring, problem solving/contextual, and followup/coordination. 

### 2.3. Statistical Analyses

The demographic, health status and diabetes characteristics of CAM practitioner users and nonusers were compared using chi-square tests for categorical variables and by logistic regression for continuous variables. All variables that were univariately associated with CAM practitioner use (*P* < 0.05) were considered as candidates for inclusion in a multiple logistic regression to predict CAM practitioner use. The multiple logistic regression was fit using a backward stepwise logistic model to determine the most parsimonious set of variables that independently predict CAM practitioner use. A stepwise multiple logistic regression yielded results identical to the backward selection model. 

## 3. Results

Use of a CAM practitioner could be ascertained for 3,337 participants. The demographic and health status characteristics of respondents of the 2009 LWDS survey are described in Table 1. The median age was 64 (IQR 57–71) and the median duration of diabetes was 7 years (IQR 3–11). Over half (54%) had diabetes-related complications, and 63.1% had a history of 2 or more either concordant or discordant comorbidities. Two hundred and fifty-eight (7.7%) of the respondents reported at least one visit to a CAM practitioner in the previous 12 months. 5.5% made more than 1 visit to a CAM practitioner (see Table 2). Only 2 CAM users reported that they had not visited their GP in the past 12 months, suggesting that the vast majority of diabetic patients were using CAM as complementary to their health care rather than as an alternative to their traditional care.

The following demographic variables were univariately associated with CAM practitioner use: female gender (*P* < 0.001), a younger age (*P* < 0.001), a higher level of educational attainment (*P* < 0.001), higher income (*P* = 0.010), and private health insurance (*P* = 0.009). Employment was also univariately associated with CAM use (*P* < 0.001) with retirees and those unable to work less likely and the unemployed more likely to visit a CAM practitioner.

No significant differences in CAM practitioner use between people with type 1, type 2 insulin requiring, or type 2 noninsulin requiring diabetes were found. Four disease characteristics were univariately associated with CAM practitioner use including the presence of neuropathy (*P* = 0.015) and history of asthma (*P* = 0.003), anxiety (*P* = 0.004), or depression (*P* = 0.002) as a comorbidity. Univariately significant health and lifestyle factors included a higher number of serves of vegetables per day (*P* < 0.001) and fish (*P* = 0.028) eaten per week. Conversely, the use of butter rather than a butter substitute or no butter was associated with CAM practitioner use (*P* = 0.020). Being sufficiently active was associated with CAM practitioner use (*P* = 0.039) relative to those that are sedentary or insufficiently active. Reporting pain on the subscale of the EQ-5D measure of health-related quality of life was univariately associated with CAM practitioner use (*P* = 0.034) as was reporting a poorer diabetes-specific quality of life (*P* = 0.004) on the ADDQoL. Participants who reported that they were less satisfied with their diabetes treatment (*P* = 0.019) as well as those less satisfied with the health care system more generally (*P* = 0.007) and those who reported that access to their main health care provider (*P* = 0.012) or access to a specialist (*P* = 0.047) was not easy were more likely to visit a CAM practitioner. In contrast, those who rated their quality of care more favourably on the PACIC were more likely to be CAM users (*P* = 0.018).

Results for the final logistic model are shown in Table 3. Twenty-one variables were univariately associated with CAM practitioner use and were entered as candidates into the multiple logistic regression model. Eight variables were retained as independent predictors of CAM practitioner use and included four socioeconomic characteristics, two health and lifestyle variables, and four health-related quality of life variables. In this diabetic patient group, younger age, more highly educated, and female gender are more likely to visit a CAM practitioner as those are with private health insurance. Diabetic patients who are CAM practitioner users can be classified as having a healthier lifestyle particularly in relation to their diet and exercise habits. Pain is an important determinant of CAM practitioner use. Those with a poorer health-related quality of life were more likely to use CAM services.

## 4. Discussion

This study examines the use of CAM practitioners using a large population-based sample of people with type 1 and type 2 diabetes. It offers a unique opportunity to investigate patterns and determinants of CAM use among this particular patient group in Australia. Our results indicate that about 8% of the LWDS respondents made at least one visit to a CAM practitioner in the previous 12 months. This level of CAM practitioner use is higher than the 4% use as reported by Armstrong et al. based on their analysis of the 2004-2005 National Health Survey database [23]. One possible explanation for this discrepancy is that the National Health Survey asked the respondents specifically about their use of CAM in the treatment or management of specific chronic conditions. In contrast, the LWDS survey did not differentiate between diabetes-specific CAM practitioner use and the use of CAM practitioners for nondiabetes conditions or general well being. As both the National Health Survey and LWDS did not record self-prescribed CAM use, the actual prevalence of CAM use among people with diabetes may be higher as studies on general population found that the use of self-prescribed CAM is common in Australia [30–32].

On the other hand, the level of CAM practitioner use we found in the LWDS sample is lower than the 17%–73% prevalence range as identified by a recent review on this topic [22]. This disparity can be explained by the fact that many previous studies had taken into consideration self-prescribed use of CAM products. The prevalence of CAM use amongst the LWDS sample should be higher if use of self-prescribed CAM products is also counted.

Our data reveal that younger age, female gender, and higher educational attainment are predictive of individuals more likely to visit CAM practitioners. This finding is broadly consistent with the profile of CAM users as identified in previous studies of general populations [33] as well as diabetes-specific populations [22]. The association of CAM practitioner use with higher income and private health insurance highlights the potential importance of the cost of CAM therapies in influencing consumption as CAM therapies or products are not currently covered by Medicare (Australia's universal health care system) or Australian Pharmaceutical Benefits Scheme (a federal government program providing subsidised prescription drugs to residents) so consumers pay the full expense when they choose these services/products.

As Table 2 demonstrates, the vast majority of this diabetes population visit CAM practitioners concurrently with their general practitioner. This reveals that people living with diabetes consider CAM therapies as a supplement rather than an alternative to mainstream treatment methods. Although the data highlights dissatisfaction with conventional health service as one of the predictors for uptake of CAM therapies, we found no evidence that CAM practitioner users receive suboptimal diabetes care and the use of CAM is not associated with HbA1c result or frequency of hypoglycaemic symptoms. However, as findings of previous studies indicated that the disclosure rate of CAM use to health care professionals remains very low [34, 35], the use of CAM alongside of conventional diabetes treatment may result in adverse reactions or drug interactions [36, 37]. This is an issue that requires further research and education. 

The present study finds that people with poorer diabetes-specific quality of life are more likely to visit a CAM practitioner. In particular, the data suggest that pain is an important determinant of CAM practitioner use among people living with diabetes. This result suggests that people are willing to try CAM therapies when conventional treatment does not help. Studies of the general population have shown that chronic pain is one of the most commonly reported conditions for which people seek out and use CAM therapies [38, 39]. The result of our study highlights the need to further investigate the role of CAM in controlling diabetes symptoms and enhancing patient's quality of life.

The results of our analysis show that CAM practitioner use is correlated with preventive and self-care behaviours, which may be part of a broader lifestyle that emphasizes on building health and resources for living. This finding is important as it highlights the potential role of CAM in health promotion and in facilitating a “structured” or lifestyle medicine approach to deal with chronic disease [40, 41]. While there is evidence that for people living with diabetes CAM is used more for improving general well being than for treating diabetes-specific conditions [10, 11, 42], researchers should not overlook the potential and benefits of CAM in prevention and management of chronic disease and in achieving integrative care for diabetes.

Several limitations of this study have to be acknowledged. First, analysis of aggregated NDSS data comparing participants of LWDS with nonparticipants indicated that individuals were more likely to participate if they were aged 50–69 years, and Indigenous Australians were less likely to participate; therefore, generalizing findings from this study to these populations must be undertaken with caution. Second, the survey only asked respondents about their visit of CAM practitioners and no information about use of self-prescribed CAM was recorded. As a result, the findings may underestimate the prevalence of CAM use among people with diabetes as there is evidence that self-prescribed CAM use is popular in Australia [30–32]. Finally, the interpretation of our findings is also limited by the fact that the health and CAM practitioner utilisation data is self-reported by the participants and is open to the effects of recall bias. However, such study limitations are outstripped by the insight gained through collecting and analysing such a large, population-based sample of people living with diabetes.

## 5. Conclusion

This paper reports the findings of a study on the prevalence, profile, and predictors of CAM practitioner use amongst Australians living with type 1 and type 2 diabetes. The study highlights that 7.7% of people with diabetes in Queensland used services of CAM practitioner alongside or as a complement to conventional health care service and preventive care. The prevalence of CAM use amongst this group of individuals would be even higher if we take into consideration self-prescribed use of CAM products.

With a surge in the prevalence of diabetes in contemporary societies and a concurrent rise in consumer interest in CAM, there is an urgent need for research to examine CAM use behaviours and the wider role of CAM in the treatment and management of diabetes given the potential risks and benefits associated with the consumption of this kind of medicine. In particular, there is a need for in-depth qualitative research on the conception and experiences of CAM use in daily management of diabetes. The frequent use of a range of practitioner-based CAM amongst people with diabetes also highlights the importance of health care providers being cognisant of CAM ingredients and encouraging an open but critical dialogue on CAM use with their patients.

## Figures and Tables

**Table 1:** Demographic and health status characteristics of respondents of the 2009 LWDS survey.

Variables	*N*	*n* (%) or median (IQR)
Prevalence of CAM practitioner use	3337	258 (7.7)
Sex	3337	
Male		1828 (54.7)
Female		1509 (45.2)
Age	3337	
18–44 year		225 (6.7)
45–59 year		909 (27.2)
60–74 years		1704 (51.1)
75+ years		499 (15.0)
Education level	3291	
University degree		449 (13.6)
Certificate/diploma/trade		1005 (30.5)
Senior high school		472 (14.3)
Year 10 and below		1365 (41.5)
Employment status	3277	
Employed (full/part time/casual/self)		1171 (35.7)
Home duties/carer		197 (6.0)
Unemployed (able to work)		66 (2.0)
Permanently ill		246 (7.5)
Retired		1597 (48.7)
Diabetes type	3337	
Type 1		144 (4.3)
Type 2: insulin requiring		615 (18.4)
Type 2: non insulin requiring		2578 (77.3)
Duration of diabetes (years)	3265	7 (3–11)
High comorbidity (2+)	3330	2101 (63.1)
Number of complications	3337	1 (0–2)
Obesity (BMI > 30)	3228	1604 (49.7)
Current smoker	3303	320 (9.7)

**Table 2:** Distribution of CAM practitioner use by visits to the general practitioner, *n* (%).

Frequency of CAM practitioner use		Visits to the GP			
	*n* (%)	None	1 to 4	5 to 11	12 or more
None	3079 (92.3)	60 (1.9)	1104 (35.9)	1258 (40.9)	657 (21.3)
1	73 (2.2)	0 (0.0)	21 (28.8)	36 (49.3)	16 (21.9)
2 to 6	125 (3.8)	2 (1.6)	44 (35.2)	50 (40.0)	29 (23.2)
7 or more	60 (1.7)	0 (0.0)	14 (23.3)	29 (48.3)	17 (28.3)

**Table 3:** Logistic model of demographic and health status characteristics of CAM practitioner users.

Variables	*N*	CAM practitioner user *n* (%) or mean ± SD	Crude OR	Crude 95% CI	Adjusted OR	Adjusted 95% CI	Adjusted *P* value
Sociodemographic							
Sex							<0.001
Male	1828	101 (5.5)	1.00		1.00		
Female	1509	157 (10.4)	1.99	1.53–2.58	1.98	1.50–2.62	
Age group							<0.001
18–44 years	225	30 (13.3)	3.18	1.80–5.62	2.30	1.25–4.23	
45–59 years	909	99 (10.9)	2.53	1.59–4.04	1.96	1.20–3.21	
60–74 years	1704	106 (6.2)	1.37	0.87–2.18	1.15	0.71–1.86	
75+ years	499	23 (4.6)	1.00		1.00		
Education level							<0.001
University degree	449	52 (11.6)	2.25	1.55–3.27	2.05	1.37–3.06	
Certificate/diploma/trade	1005	93 (9.2)	1.75	1.28–2.41	1.84	1.32–2.57	
Senior high school	472	37 (7.8)	1.46	0.97–2.20	1.48	0.96–2.28	
Year 10 and below	1365	75 (5.5)	1.00		1.00		
Health insurance							0.011
Private	1712	155 (9.1)	1.41	1.09–1.83	1.44	1.09–1.92	
Not private	1551	102 (6.6)	1.00		1.00		
Health and lifestyle							
Vegetable consumption (per serve per day)		3.3 ± 1.3	1.18	1.08–1.30	1.17	1.06–1.29	0.002
Exercise							
Sufficiently active	1568	138 (8.8)	1.31	1.01–1.69	1.34	1.02–1.76	0.035
Insufficiently active	1718	118 (6.9)	1.00		1.00		
Quality of life							
EQ-5D pain subscale							0.002
Extreme pain or discomfort	196	20 (10.2)	1.68	1.01–2.80	1.99	1.15–3.44	
Moderate pain or discomfort	1685	142 (8.4)	1.36	1.04–1.79	1.66	1.23–2.23	
No pain or discomfort	1421	90 (6.3)	1.00		1.00		
ADDQoL AWI (per unit)		−1.8 ± 1.8	0.91	0.85–0.97	0.92	0.86–0.99	0.032

## Glossary

ADDQoL: Audit of Diabetes Dependent Quality of Life
CAM: Complementary and alternative medicine
LWDS: Living with Diabetes Study
NDSS: National Diabetes Services Scheme
PACIC: Patient Assessment of Chronic Illness Care.
